# Floods of Egypt’s Nile in the 21st century

**DOI:** 10.1038/s41598-024-77002-8

**Published:** 2024-11-07

**Authors:** Ahmed Badawy, Mohamed Sultan, Karem Abdelmohsen, Eugene Yan, Hesham Elhaddad, Adam Milewski, Hugo E. Torres-Uribe

**Affiliations:** 1 Department of Geological and Environmental Sciences, Western Michigan University, Kalamazoo, MI 49008 USA; 2 Geology Department, Faculty of Science, Tanta University, Tanta, 31527 Egypt; 3 School of Sustainability, Arizona State University, Tempe, AZ 85287 USA; 4 Environmental Science Division, Argonne National Laboratory, Lemont, IL 60439 USA; 5 Department of Geology, University of Georgia, Athens, GA 30602 USA

**Keywords:** Climate change, Hydrology, Climate sciences, Environmental sciences, Hydrology, Natural hazards

## Abstract

Extreme precipitation and flooding events are rising globally, necessitating a thorough understanding and sustainable management of water resources. One such setting is the Nile River’s source areas, where high precipitation has led to the filling of Lake Nasser (LN) twice (1998–2003; 2019–2022) in the last two decades and the diversion of overflow to depressions west of the Nile, where it is lost mainly to evaporation. Using temporal satellite-based data, climate models, and continuous rainfall-runoff models, we identified the primary contributor to increased runoff that reached LN in the past two decades and assessed the impact of climate change on the LN’s runoff throughout the twenty-first century. Findings include: (1) the Blue Nile subbasin (BNS) is the primary contributor to increased downstream runoff, (2) the BNS runoff was simulated in the twenty-first century using a calibrated (1965–1992) rainfall-runoff model with global circulation models (GCMs), CCSM4, HadGEM3, and GFDL-CM4.0, projections as model inputs, (3) the extreme value analysis for projected runoff driven by GCMs’ output indicates extreme floods are more severe in the twenty-first century, (4) one adaptation for the projected twenty-first century increase in precipitation (25–39%) and flood (2%-20%) extremes is to recharge Egypt’s fossil aquifers during high flood years.

Extreme precipitation and drought events are becoming more common in many places worldwide. In arid and semi-arid regions, understanding these extremes’ frequency, intensity, distribution, and driving forces is more necessary than ever while creating sustainable methods for capturing and managing these additional water resources. The impact of global warming on hydrological systems has been the subject of study and discussion for the last twenty years^1–5^. Precipitation is one of the primary climate variables researchers investigate^6^. The long-term global trends in average precipitation, as observed by meteorological satellites, indicate an increase in the global mean precipitation with global warming (1–3% per kelvin from 1987 to 2006)^7^. An increase in heavy (2–10 mm/hour) and extremely heavy precipitation (> 10 mm/hour) events and a reduction in moderate to light events (0.1–2.0 mm/hour) are projected based on an examination of numerous global and regional climate models^8^. Global climate models predict a dramatic increase in flood frequency and associated geohazards^9,10^ by the close of the twenty-first century, especially in the Indian Peninsula, Southeast Asia, the northern region of the Andes, and eastern Africa (Nile River source areas)^11,12^.

The Nile River stretches approximately 6700 km, making it the longest river globally; it originates south of the equator, travels across 11 countries, and flows northward through northeastern Africa before draining into the Mediterranean Sea^13^. The Blue Nile and White Nile are the two primary tributaries of the Nile River, along with a third, less prominent tributary, the Atbara. The White Nile journey begins in south Rwanda within the Great Lakes region, continues northward, traversing via Tanzania, Lake Victoria, and Uganda, and finally reaches South Sudan. The Blue Nile begins its journey from Lake Tana in Ethiopia, eventually joining the flow into Sudan. Both rivers converge at Khartoum and run northward through Sudan and Egypt, where they enter Lake Nasser (LN) and flow further downstream to reach the Nile Delta before draining into the Mediterranean Sea.

The annual rainfall in the Blue Nile subbasin (BNS) varies from 1000 mm/year in the northeast to 2000 mm/year in the southeast^13,14^, with an overall average of 1346 mm/year, making it the highest among all Nile subbasins^13^. The annual rainfall in the Ethiopian highlands varies from 1200 to 1800 mm/year, averaging approximately 1227 mm/year^15^. Precipitation in the Equatorial Lakes region ranges between 700 and 1700 mm/year across two wet seasons (March to May and August to December), with an average of approximately 1200 mm/year^15^. The Atbara subbasin (AS) receives rainfall from 90 to 1300 mm annually, with an average annual rainfall of about 553 mm/year, the lowest among the other Nile subbasins^13^.

The total runoff in the Nile River basin is around 161 km^3^/year^16^. The average runoff measured at Aswan’s High Dam is approximately 84 km^3^/year^17^, 57 to 59 % of which comes from the Blue Nile, 28% from the White Nile, and 13 to 14 % from the Atbara River based on stream flow data from 1900 to 1950^18,19^.

We selected as our study areas: (1) the source areas of the BNS, White Nile subbasin (WNS), and AS, where the BNS, WNS, and AS are the primary Nile subbasins (Fig. 1), and (2) the Tushka Lakes (TLs) as well as LN that receive water from the source areas via the White Nile and Blue Nile (Figs. 1, 2). Twice within the past two decades (1998–2003, 2019–2022), high precipitation over the source areas of the Nile River flowed downstream and flooded large areas in Sudan’s capital, Khartoum^20^, and continued its journey to the southern borders of Egypt, got impounded behind the Aswan High Dam and filled LN. The overflow was diverted into major depressions within the limestone plateau, bounding the Western Desert from the west, forming the Tushka Lakes (TLs; Fig. 2). If not for the Aswan High Dam and the spillways to the Tushka depressions, the excess LN waters would have devastated Egypt’s population as it did in Sudan.

**Fig. 1:**
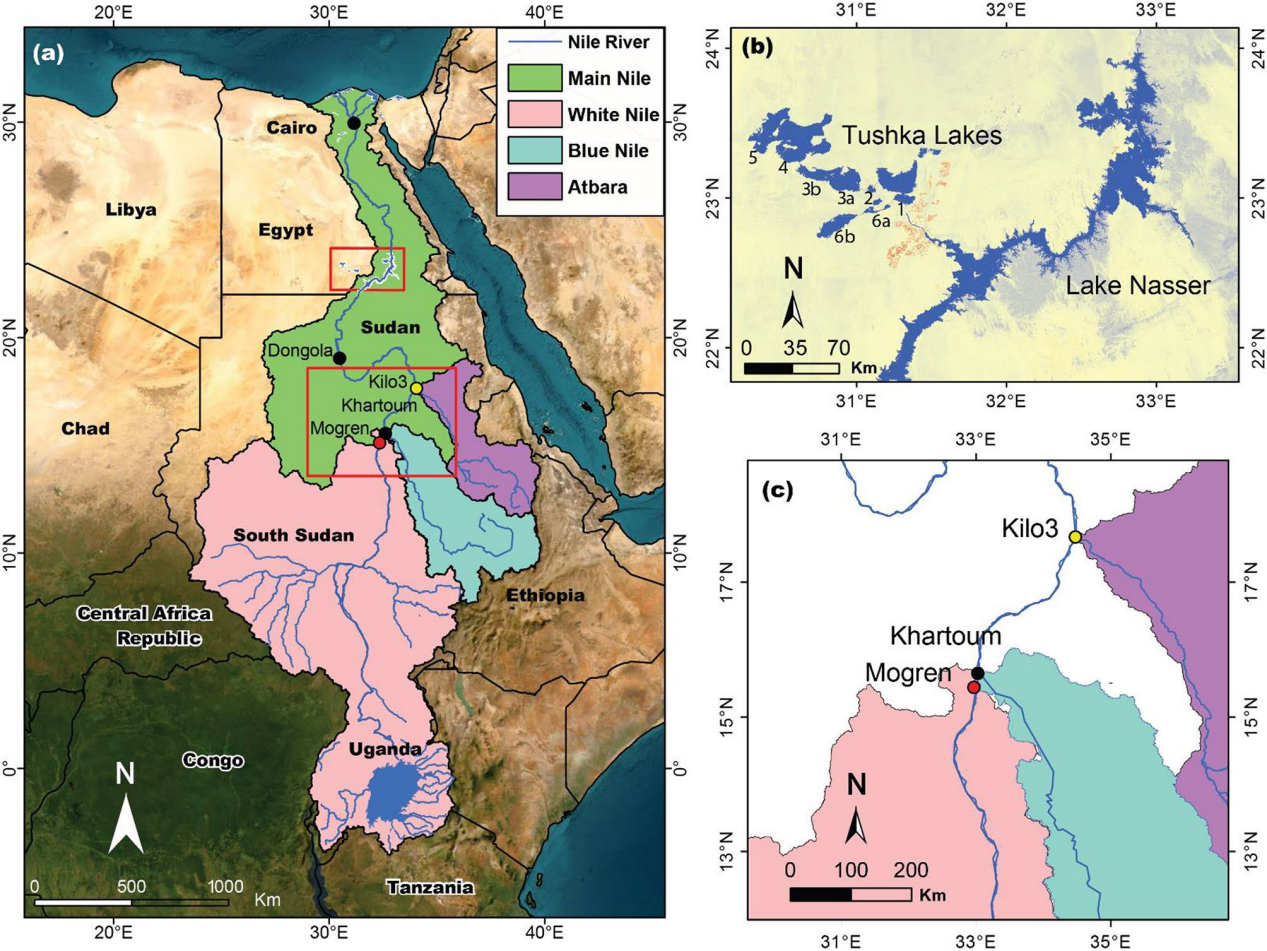
(**a**) Location map of the Nile Basin and its White Nile subbasin (WNS), Blue Nile subbasin (BNS), and Atbara Subbasin (AS), (**b**) enlargement of the areas covered by the upper red box covering Lake Nasser (LN) and Tushka Lakes (TLs), (**c**) enlargement of the areas covered by the lower red box showing the locations of three-gauge stations at the outlets of the WNS, BNS, and AS.

**Fig. 2:**
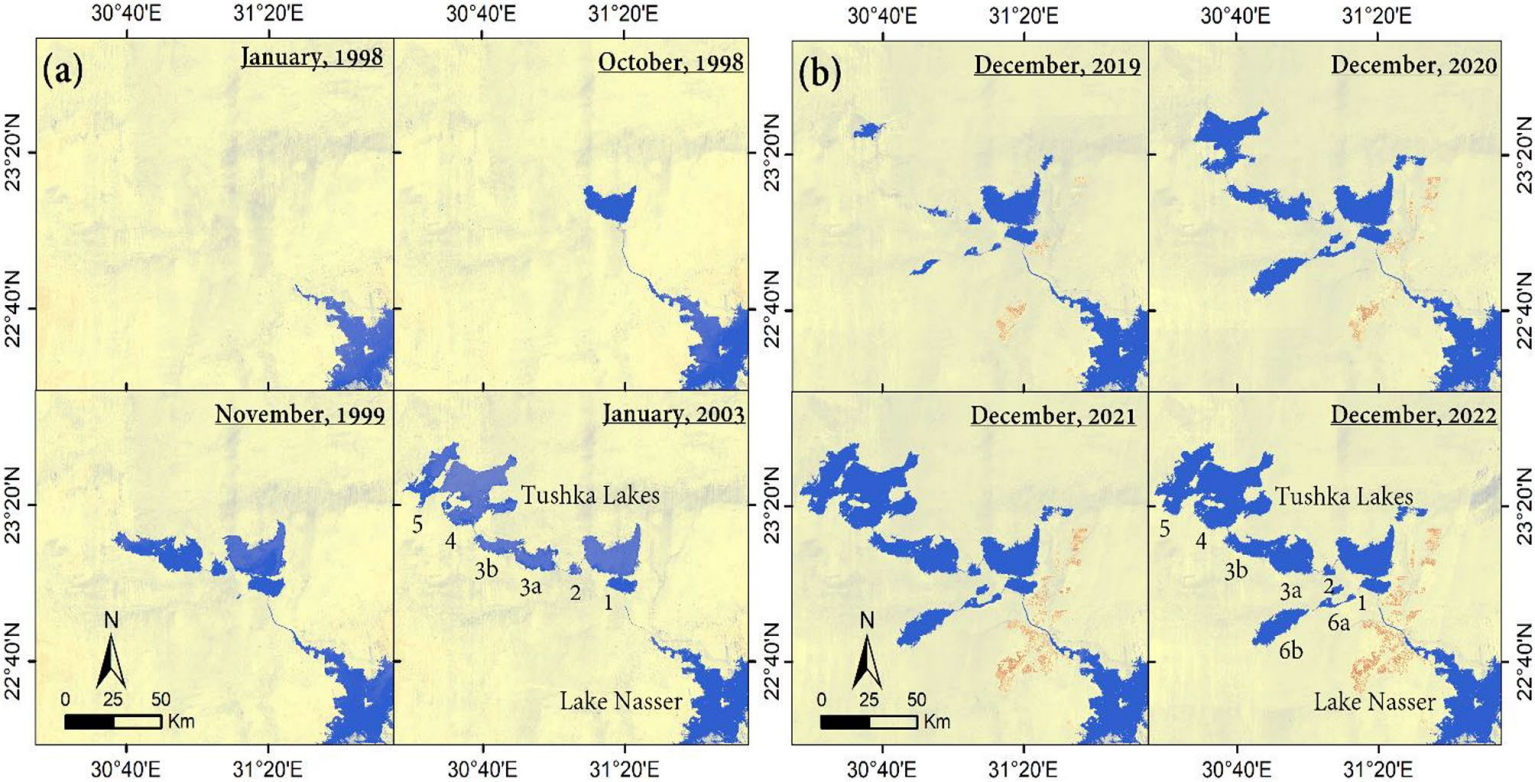
(**a**) Temporal Landsat images (1998/2003), (**b**) Temporal Sentinel-2 images (2019/2022) showing progressive filling of the Tushka Lakes (TLs) by excess Lake Nasser (LN) waters.

A surface water model calibrated by adjusting routing parameters against lake stages (1998–2002) revealed the TLs received about 27.4 km^3^ of water from late 1998 to early 2002, 39% of which was lost to direct evaporation in this period^21^. This fate awaits the waters in the depressions from the 2019–2022 recent flooding event. This is happening while Egypt desperately needs additional fresh water supplies, and its largest aquifer in the Western Desert, the Nubian Sandstone Aquifer System (NSAS), is being depleted due to the extraction of fossil water for irrigation. This aquifer was replenished throughout previous wet Pleistocene climatic periods^22–24^. Utilizing temporal Gravity Recovery and Climate Experiment (GRACE) and GRACE Follow-On (GRACE-FO), hereafter referred to as GRACE, the Dakhla subbasin has been depleted at an average rate of -2.28 ± 0.08 km^3^/year^25^ from April 2002 to September 2019.

Three objectives were sought: (1) identify the nature of excessive precipitation over the source areas of the Blue Nile, the White Nile, and Atbara that caused extreme flooding in downstream countries during 1998–2003 and 2019–2022; (2) identify the timing and frequency of the precipitation events that caused the last two extreme flooding in downstream countries and throughout the twentieth century; and (3) predict the impacts of global warming on the runoff reaching the downstream countries for the period 2020–2100.

The objectives were accomplished by conducting the following tasks: (1) examine the temporal variations in Gravity Recovery and Climate Experiment Terrestrial Water Storage (GRACE_TWS_), precipitation, and runoff from the Blue Nile, the White Nile, and Atbara to identify the subbasin(s) responsible for excessive flooding and runoff reaching downstream countries; (2) develop and calibrate a continuous rainfall-runoff model for the subbasin(s) causing the excessive flooding and runoff and use the model(s) to evaluate dynamic changes in runoff and identify the timing and frequency of the precipitation patterns that caused the 1998–2003 and 2019–2022 flooding events in downstream countries as well as similar events throughout the twentieth century; (3) downscale and bias-correct results from three global circulation models (GCMs), Community Climate System Model version 4 (CCSM4), the Hadley Centre Global Environmental Model version 3 (HadGEM3), and Geophysical Fluid Dynamics Laboratory (GFDL)’s CM4 model (GFDL-CM4.0), hereafter referred to as the three GCMs, over the BNS; (4) use the calibrated model with downscaled outputs of CCSM4, HadGEM3, and GFDL-CM4.0 as model input to simulate climate change effects on the runoff reaching the downstream countries for the period 2020–2100; and (5) conduct extreme value and uncertainty analysis of projected precipitation and runoffs to predict frequency and severity of flooding extremes in the twenty-first century and quantify the associated model uncertainty.

## Results

Findings from the application of each of the main five tasks listed above and described in detail in the “Methods” are provided in this section.

### Identify the primary source (subbasin) of increased Nile waters that reached LN in 2019–2022

We examined the contributions of the individual subbasins over an extended period (1900–1999) and investigated whether the BNS was the main contributor to the 1998–2003 and the 2019–2022 flooding events. Our analysis of the stream flow data from the Mogren (WNS), Khartoum (BNS), and Kilo3 (AS) gauge stations (Fig. 1c) for the period 1900 to 1999 revealed that the average contribution of the BNS, WNS, and AS are 60.3%, 25.5%, and 14.2%, respectively.

We monitored the filling of the TLs from October 2019 until December 2022 and observed a progressive increase in TLs water storage (maximum storage: 2019 is 7.38 km^3^; 2020 is 24.57 km^3^; 2021 is 38.58 km^3^; 2022 is 57.58 km^3^). For each of the four years, the storage capacity grew from September, which signaled the start of the flooding season, until December and remained at near-constant levels until the following flooding season (Supplementary Fig. 1 and Supplementary Table 1).

**Fig. 3:**
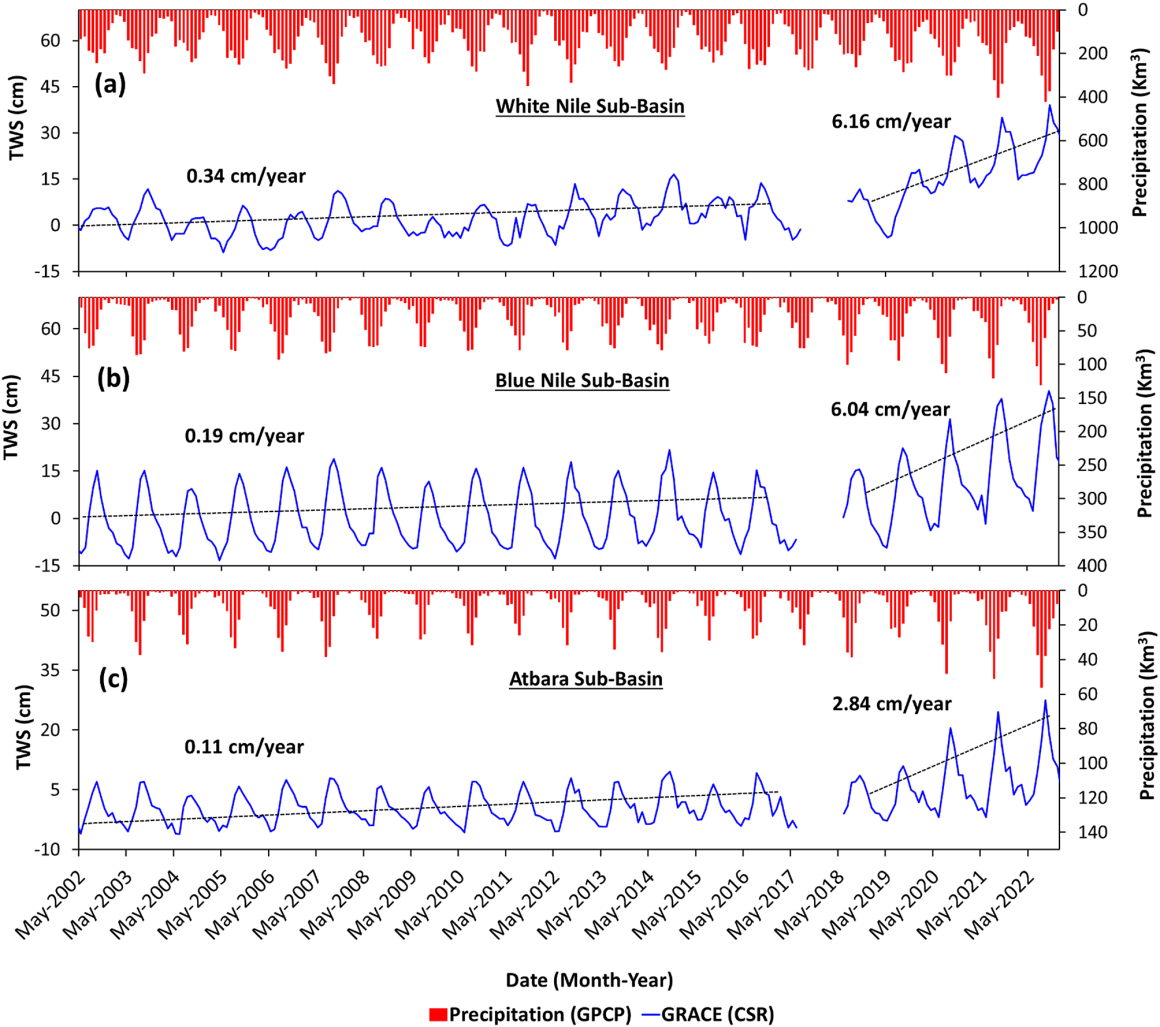
Comparison between the monthly time series of the GRACE_TWS_ and GPCP precipitation over the Nile River Subbasins. (**a**) WNS (**b**), BNS, and (**c**) AS. The gap area shows the missing data between the GRACE and GRACE-FO mission from July 2017 to May 2018.

The diversion of LN waters to the TLs (2019–2022) was found to be contemporaneous with an increase in precipitation and GRACE_TWS_ over the WNS, BNS, and AS. The Average Annual Precipitation (AAP) over the WNS, BNS, and AS amounted to 1606 km^3^, 344 km^3^, and 99 km^3^, respectively, from 2002 to 2018. It increased in the following years (2019–2022) in all three subbasins (WNS 1921 km^3^, BNS 420 km^3^, and AS 157 km^3^) by 19.6%, 22%, and 58.5%, respectively. The GRACE_TWS_ values over each of the subbasins rose during the period 2019 to 2022 (WNS: 6.16 cm/year, BNS: 6.04 cm/year, and AS: 2.84 cm/year) compared to the earlier period 2002–2018, where the trends were 0.34 cm/year, 0.19 cm/year, and 0.11 cm/year, respectively (Fig. 3). All three subbasins witnessed increased precipitation and GRACE_TWS_ values contemporaneous with filling of the TLs, suggesting a causal effect.

Since the three subbasins witnessed an increase in precipitation and GRACE_TWS_ over the period 2019 to 2022, and the BNS contributes some 60.3% of the total Nile water reaching Egypt, we conclude the increase in rainfall over the BNS is the primary source of the increased Nile waters that reached LN in 2019–2022.

### Construct, calibrate, and validate a continuous rainfall-runoff model over the BNS

The Blue Nile watershed was divided into eight subbasins, with an outlet at the location of each of the selected gauge stations (Shamboo, Asosa, Sudan Border, Kessie, Merawi, Roserires, Sennar and Khartoum; Supplementary Fig. 2a,b). The subbasins were broken down into Hydrologic Response Units (HRUs), totaling 698, each distinguished by distinct soil sets, land use, and slope types. Soil types included Vertisols, Aridisols, Ultisols, Alfisols, Inceptisols, and Entisols, with Vertisols and Inceptisols being the dominant soil types (Supplementary Fig. 2c); land cover classes included Baresoil, Sparse Vegetation, Herbaceous Vegetation, Shrubs Covered Area, Tree Covered Area, Grass Land and Crop Land (Supplementary Fig. 2d).

The model was calibrated using SWAT-CUP2012 software (Soil & Water Assessment Tool). Sixteen parameters were considered (Supplementary Table 2); only eight were significant based on the global sensitivity analysis results and selected for model calibration. The t-value for the sensitivity test ranged from 3 to – 35 (Supplementary Table 3). The rankings for the flow parameters are provided in Supplementary Table 3, while the fitted values for the most sensitive parameters are found in Supplementary Table 4.

**Fig. 4:**
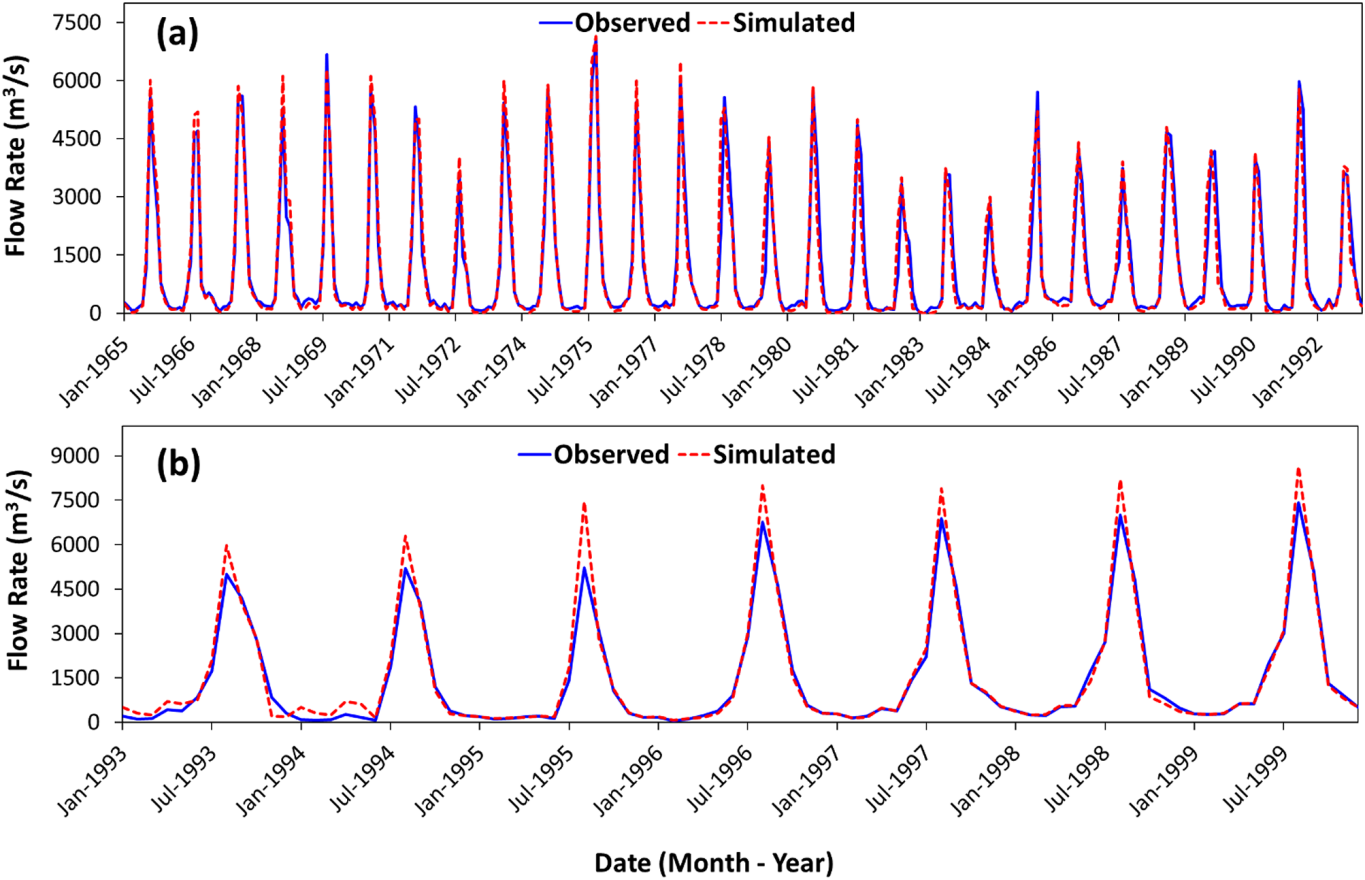
Monthly observed and simulated stream flow hydrographs for (**a**) the calibration period (1965–1992) (**b**) the validation period (1993–2000) at the Khartoum gauge station.

Adjustments were made to the starting values of the selected parameters to enhance the consistency of model simulations with the measured flow data. The model underwent calibration and validation by comparing the simulated and observed monthly flow data at the Khartoum station from 1965 to 1992 (Fig. 4a) and 1993 to 2000, respectively (Fig. 4b). The simulated monthly flow agreed^26^ with the observed values during the calibration timeframe (NSE: 0.80, R^2^: 0.85; and PBIAS: 5.06%) and the validation timeframe (NES: 0.83; R^2^: 0.87; and PBIAS: 3.50%).

### Downscale and bias-correct CCSM4, HadGEM3, and GFDL-CM4.0 outputs over the BNS

**Fig. 5:**
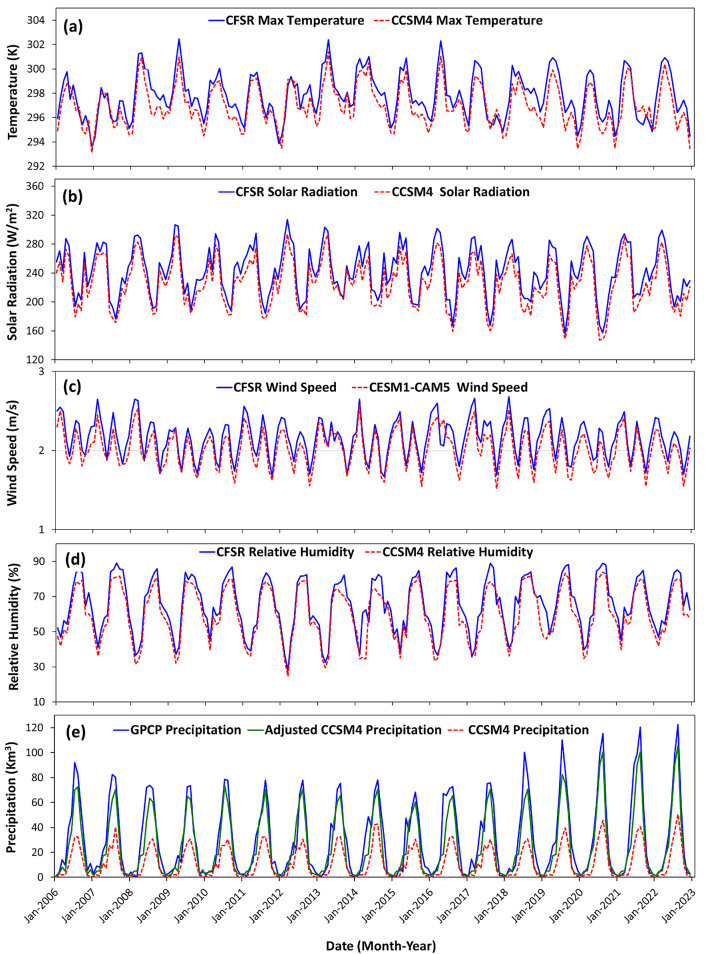
Comparison of average monthly (**a**) maximum temperature, (**b**) solar radiation, (**c**) wind speed, (**d**) relative humidity, and (**e**) precipitation extracted from CCSM4, CESM1-CAM5, CFSR and GPCP datasets over the Blue Nile subbasin from 2006 to 2022.

An agreement, in terms of both magnitude and phase, was noted among the outputs of the modeled parameters from the three GCMs (CCSM4, HadGEM3, GFDL-CM4.0) and the Community Earth System Model—Community Atmosphere Model (CESM1-CAM5), and the measured parameters from Climate Forecast System Reanalysis (CFSR) database. The following parameters were considered: maximum or minimum air temperature, solar radiation, wind speed, relative humidity, and precipitation (Fig. 5a–e) during the overlapping period (2006 to 2023). A good correspondence is observed in phase and magnitude between the modeled (CCSM4) and observed parameters, except for precipitation. The temporal variations in the CCSM4 precipitation values are in phase with, but consistently lower in magnitude than the Global Precipitation Climatology Project (GPCP) values. Upon applying the bias correction to the CCSM4 precipitation, we observe an agreement between the observed GPCP average monthly precipitation and the adjusted average monthly CCSM4 for the period 2006–2022 (Fig. 5e). The correction function derived from the reference period was applied to rectify the bias in the precipitation data of CCSM4 from 2020 to 2100. Similarly, a good correspondence was observed between the modeled (from the three GCMs) and observed parameters, except for precipitation. Accordingly, the bias correction was restricted to rainfall.

### Use the outputs of down-scaled and bias-corrected GCMs throughout the twenty-first century as inputs to the calibrated rainfall-runoff model

**Fig. 6:**
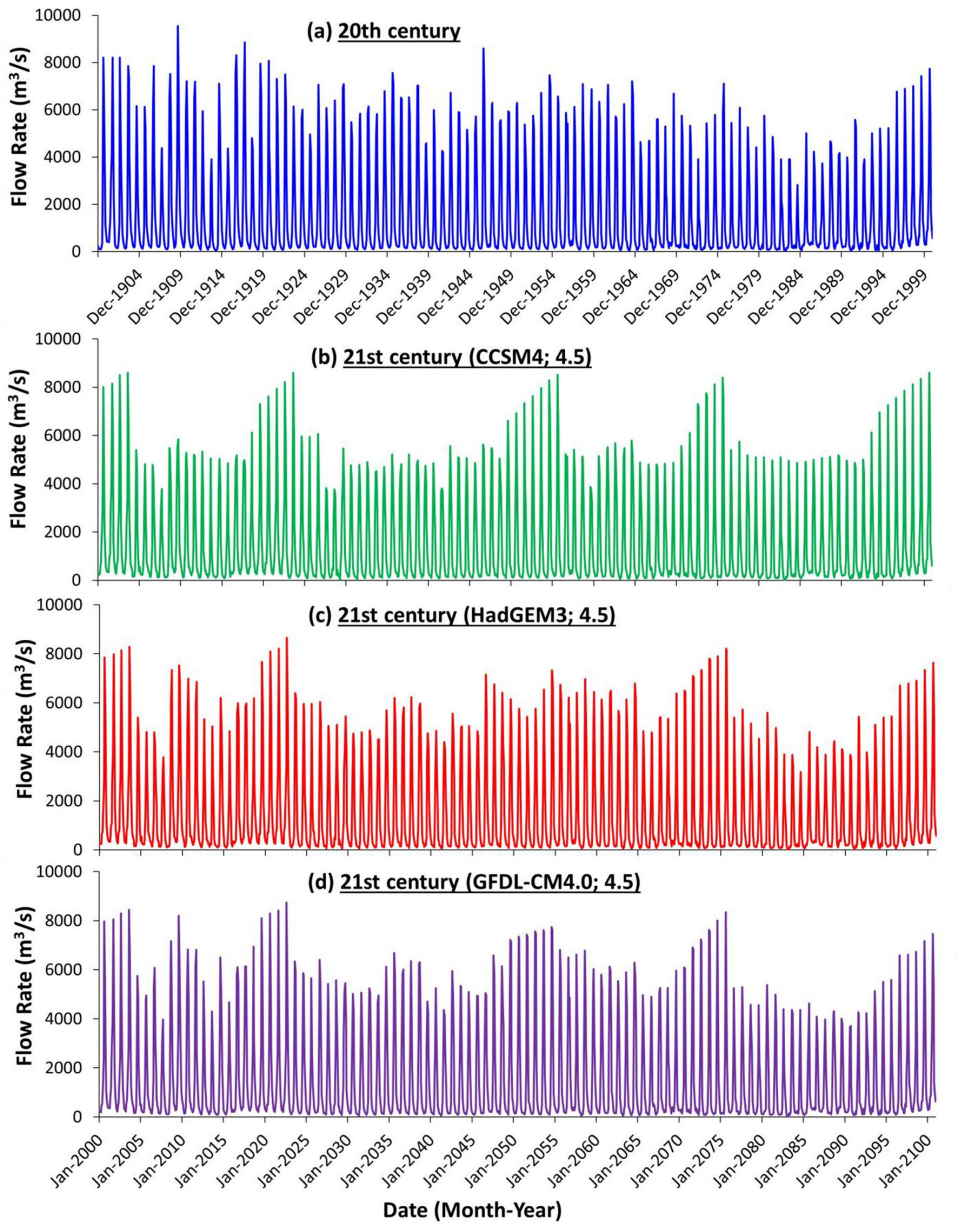
Comparison between observed and predicted flow rates from continuous rainfall-runoff model at the Khartoum gauge station in the twentieth and twenty-first centuries based on the RCP4.5 pathway from the selected three GCMs. (**a**) observed flow rates during the twentieth century. (**b**) predicted flow rates during the twenty-first century based on the CCSM4 model parameters. (**c**) predicted flow rates during the twenty-first century based on the HadGEM3 model parameters. **d**) predicted flow rates during the twenty-first century based on the GFDL-CM4.0 model parameters.

We compared the observed flow rates at the Khartoum station in the twentieth century (Fig. 6a, Supplementary Fig. 3a) with the estimated twenty-first century flow rates (Fig. 6b–d, Supplementary Fig. 3b–d). Those were calculated using outputs of down-scaled and bias-corrected CCSM4, HadGEM3, and GFDL-CM4.0 parameters as inputs to the calibrated continuous rainfall-runoff model and applying the Representative Concentration Pathway (RCP) 4.5 (Fig. 6) and (RCP) 8.5 (Supplementary Fig. 3) pathways. The comparison reveals a significant increase in the flow rate in the twenty-first century compared to the twentieth century. The mean flow rate in the twentieth century was 6061 m^3^/s, whereas, in the twenty-first century, it was 6897 m^3^/s under the CCSM4 model (RCP 4.5) pathway and 7088 m^3^/s under the RCP 8.5 pathway. The mean flow rate for the HadGEM3 model is 6619 m3/s (RCP 4.5) and 6643 m^3^/s (RCP 8.5), and for the GFDL-CM4.0 model, the mean flow rate is 6925 m^3^/s (RCP 4.5) and 6901 m^3^/s (RCP 8.5).

We examined the flow rates at the Khartoum station in search of patterns characterizing the 1998–2003 and 2019–2022 events to assist in the search for similar flow patterns in the twenty-first century rates. The twenty-first century flow rates were estimated using outputs of down-scaled and bias-corrected CCSM4, HadGEM3, and GFDL-CM4.0 parameters as inputs to the calibrated SWAT model. We observed progressively increasing flow rates in 7 to 8 consecutive years, including the event years. For example, under the CCSM4 model, the peak flow for the first event (1998–2003) increased from 7002 m^3^/s in 1998 to 8600 m^3^/s (RCP 4.5) and 9400 m^3^/s (RCP 8.5) in 2003 (Fig. 6a,b, and Supplementary Fig. 3b). For the second event (2019–2022) under the CCSM4 model, the peak flow rate increased from 7305 m^3^/s (2019) to 8222 m^3^/s (2022) and 8592 m^3^/s in 2023 (RCP 4.5) (Fig. 6b) and from 8861 m^3^/s in 2019 to 9952 m^3^/s in 2022 and 9999 m^3^/s in 2023 (RCP 8.5) (Supplementary Fig. 3b). We recognize three similar flow rate patterns for the remaining periods of the twenty-first century for the three GCMs (4.5 and 8.5 pathways). For example, for the CCSM4 model (4.5 pathway), the flow rate for the first of the three periods increased from 6602 m^3^/s (2049) to 8523 m^3^/s (2055), from 5557 m^3^/s (2070) to 8407 m^3^/s (2075) in the second period and from 6120 m^3^/s (2093) to 8604 m^3^/s (2100) in the third period (Fig. 6b–d, Supplementary Fig. 3b–d).

### Extreme value and uncertainty analysis of precipitation and stream flow data in the twentieth and twenty-first centuries

**Fig. 7:**
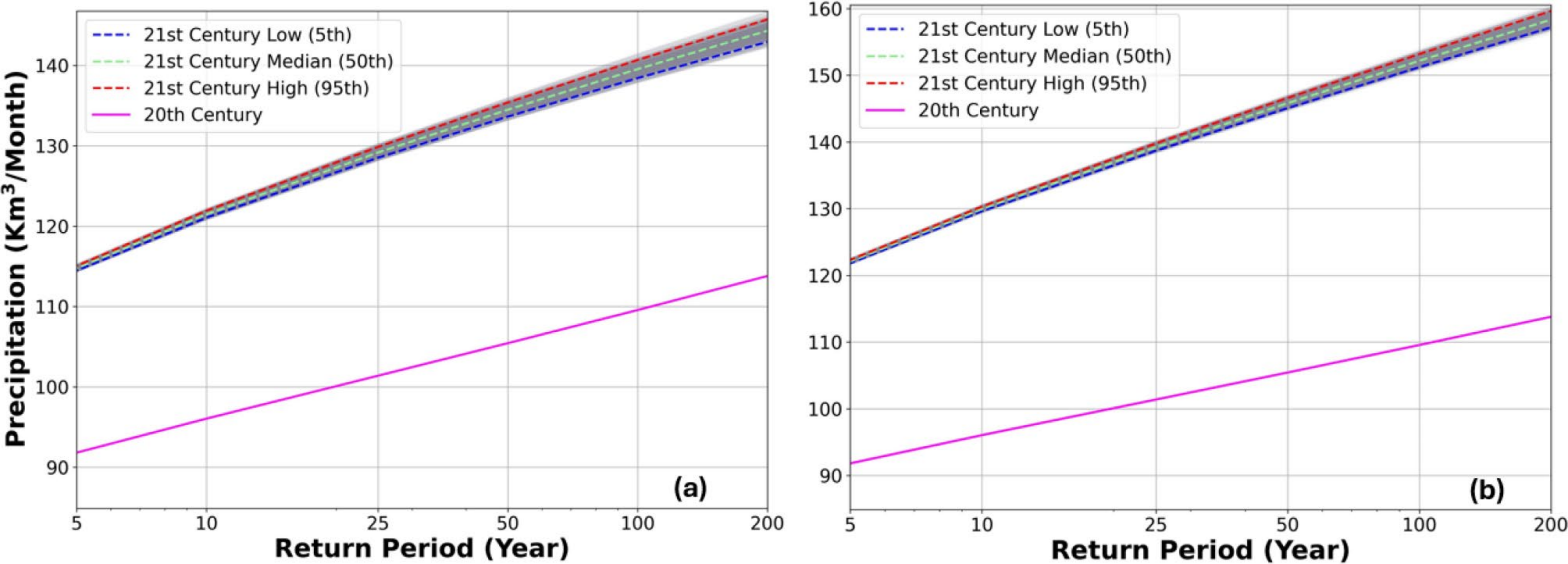
Predicted precipitation intensities with GLO distributions for extremes at return periods of 5, 10, 25, 50, 100, and 200 years in the twentieth and twenty-first centuries under (**a**) RCP 4.5 and (**b**) RCP 8.5 scenarios. The uncertainties of precipitation predictions for the twenty-first century are quantified by 500 GLO distribution curves (grey lines), and the upper and lower bounds (red and blue dashed lines) are defined by 95th and 5th percentiles, respectively.

The results of extreme value analysis with seven statistical distributions suggest that Generalized Logistic distribution (GLO) fits the best for annual maximum precipitation monthly data, and Log-Pearson Type III (LP3) distribution fits well with annual maximum stream flow monthly data based on the criterion of L-moment parameters 3 (kurtosis) and 4 (skewness)^27^. The derived GLO distributions provide estimates of precipitation intensity as a monthly duration at return periods of 5-, 10, 25-, 50-, 100-, and 200-year extreme events (Fig. 7a,b). The precipitation intensity in the twentieth century is 92, 96, 101, 105, 110, and 114 km^3^/month for 5-, 10-, 25-, 50-, 100-, and 200-year events, respectively. For the twenty-first century, the precipitation intensity is predicted to increase by 25%, 26%, 27%, 28%, 27%, and 27% under the RCP 4.5 pathway and 33%, 35%, 37%, 38%, 39%, and 39% under the RCP 8.5 pathway for the corresponding extreme events, respectively, based on estimated median values as shown in Fig. 7a,b. The uncertainties of precipitation projections due to variation among GCM models and their outputs for the twenty-first century were quantified by ensembles of 500 annual maximum series (AMS) randomly sampled from the output of the three GCMs. The uncertainties represented by 500 GLO distribution curves are depicted in Fig. 7a,b, and the 95th and 5th percentiles define their upper and lower bounds, respectively. The uncertainty range, an interval between the upper and lower bounds, is from 1 to 3 km^3^/month for RCPs 4.5 and 8.5 (increasing with higher return periods or less frequent extremes), much smaller than the increase in precipitation intensity.

**Fig. 8:**
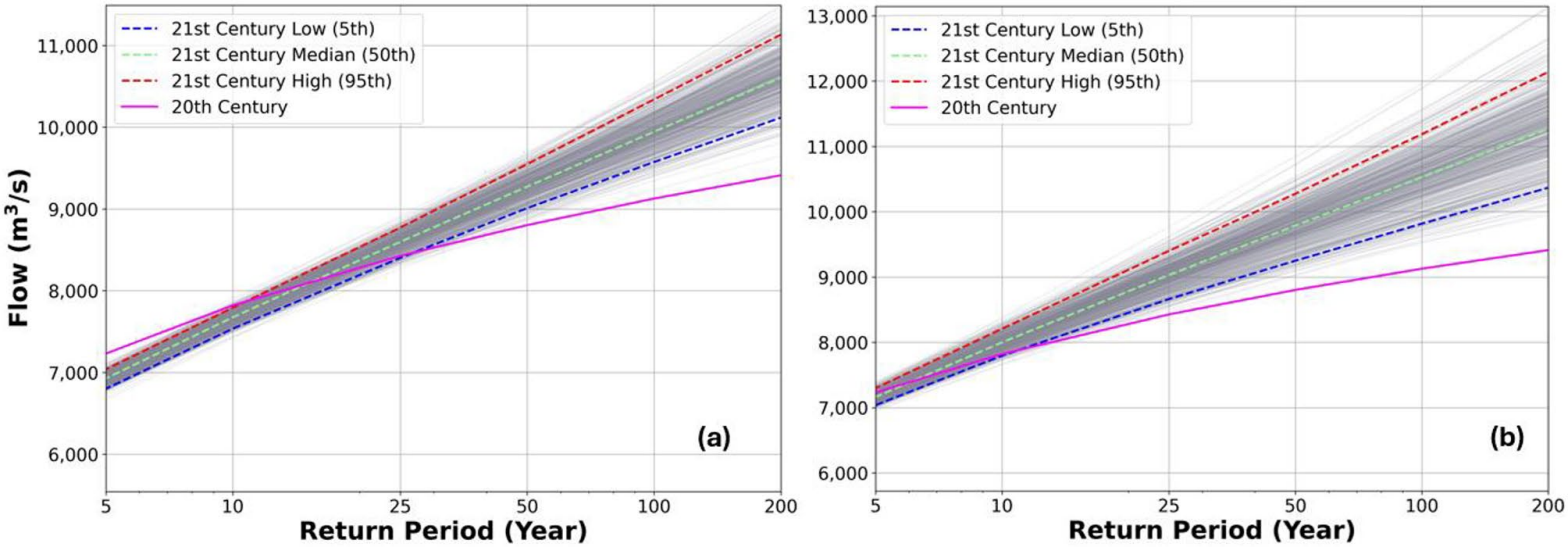
Predicted peak stream flow extremes with LP3 distributions at return periods of 5, 10, 25, 50, 100, and 200 years in the twentieth and twenty-first centuries under (**a**) RCP 4.5 and (**b**) RCP 8.5 scenarios. The uncertainties of peak stream flow predictions for the twenty-first century are quantified by 500 LP3 distribution curves (grey lines), and the upper and lower bounds (red and blue dash lines) are defined by 95th and 5th percentiles, respectively.

The LP3 distributions derived from annual maximum monthly stream flows estimate the annual peak flows in the twentieth century at 7230, 7820, 8426, 8801, 9126, and 9411 m^3^/s for the return periods of 5-, 10-, 25-, 50-, 100-, and 200-years, respectively (Fig. 8a,b). The uncertainties of stream flow projections for the twenty-first century due to output variation of the hydrologic model propagated from GCM model variations were quantified; this was accomplished using ensembles of 500 AMS randomly sampled from stream flows predicted by hydrologic simulations driven by the three GCM projections. Although the flow uncertainty propagated from GCM model variations is much greater than precipitation uncertainty, stream flows still show a statistically significant increase for the 25-year events or above for RCP 4.5 and 10-year events or above for RCP 8.5. Based on the predicted median values of stream flows for the twenty-first century, the stream flows are likely expected to increase by 2%, 5%, 9%, and 13%, corresponding to 25-, 50-, 100-, and 200-year events, respectively, under RCP 4.5 and 2%, 7%, 11%, 15% and 20% for 10-, 25-, 50-, 100-, and 200-year events, respectively, under RCP 8.5 (Fig. 8a,b).

### Applications and limitations

We developed a calibrated rainfall-runoff model for the BNS. We used the outputs of downscaled and bias-corrected variables from the three GCMs as inputs to the model to gain insights into the intensity and frequency of floods in the downstream countries and the optimum ways to address the projected twenty-first century scenarios. The BNS was selected because it is the primary contributor (~ 60%) to the Nile waters reaching Egypt.

Our findings suggest a rise in the intensity of precipitation and floods, especially for extreme events at the return periods of 10 years or more, during the twenty-first century. The Aswan High Dam, which can hold up to 150 km^3^, mitigated the flood and drought conditions in the twentieth and twenty-first century scenarios to a large extent. However, LN could not handle the excess flood waters of 1998–2003 and 2019–2022; those had to be diverted to the Tushka depressions and were left to evaporate. Our twenty-first century predictions could be enhanced by extending our modeling over the other two Nile subbasins, the WNS and the AS, to model the overall flow toward Egypt. In this case, the flow will be calibrated against stream flow data from the Dongola station (Fig. 1) and the twenty-first century runoff estimated at this location. The extreme value analysis will provide estimates of total peak stream flows for extreme events in the twenty-first century.

Given the projected increases in precipitation (25%-39%) and flooding extremes (2%-20%) in the twenty-first century, solutions must be made to protect Egypt’s population against the projected excessive flooding events, minimize their adverse effects, and explore adaptations that could replace the catastrophic consequences of climate change with beneficial and sustainable development opportunities upon implementation. It has been demonstrated that the dam’s operational rules could be modified (using aridity indices) to adapt to climate change-related streamflow alterations while maintaining the supply for downstream water use^28^. Complementary engineering scenarios were also suggested to divert excess LN waters to surrounding natural depressions where the NSAS crops out to recharge its fossil waters in high flood years^29^. The study utilized hydrodynamic modeling (RiverFlow2D) to test optimum release scenarios of excess LN waters in surrounding lowlands and partition the released waters into runoff, recharge into the NSAS, and evaporation^29^. They determined that the optimum scenario was the release of excess LN waters comparable to that of the 2019–2022 event into the depressions south of the TLs floored by NSAS outcrops. This scenario produced the highest infiltration, the least losses to evaporation, and minimal encroachment on infrastructure. The simulated infiltration amounted to 74.3% of the released waters, the losses to evaporation to 20.1%, and the surface runoff to 5.6% after 4.5 years from the water’s release. The projected increase in the BNS flooding events in the twenty-first century and the solutions mentioned above for utilizing those resources are incremental steps that pave the way towards developing comprehensive and sustainable management strategies for the Nile River water resources in the twenty-first century.

## Methods

Our methodology encompasses five main steps to accomplish the following: (1) identify the primary source (subbasin) of the increased Nile waters that reached LN in 2019–2022, (2) construct a rainfall-runoff model over the Nile subbasin(s) that largely contributed to the excess runoff during the 1998–2003 and 2019–2022 events, and calibrate and validate the model against stream flow data, (3) downscale, and bias-correct outputs of the three GCMs over the selected Nile subbasin(s), (4) use the outputs of the down-scaled and bias-corrected GCMs throughout the twenty-first century as inputs to the calibrated rainfall-runoff model to estimate the runoff at the outlet of the selected Nile subbasin in the twenty-first century, and (5) conduct extreme value and uncertainty analysis of stream flow data in the twentieth and twenty-first centuries.

### Identify the primary source (subbasin) of increased Nile waters that reached LN in 2019–2022

We examined precipitation and GRACE time series over the WNS, BNS, and AS to identify differences in their temporal precipitation patterns and to examine GRACE’s response to wet (2019–2022) and dry (2003–2018) periods. We also extracted the temporal variations in LN and TLs surface water levels and volumes to investigate the correspondence between those datasets and the precipitation and GRACE time series over the Nile subbasins.

The Average Annual Precipitation (AAP) and time series across the Nile subbasins were extracted from the GPCP datasets version 2.3^30^ (https://psl.noaa.gov/data/gridded/data.gpcp.html). The GPCP is a monthly global gridded (2.5° × 2.5°) precipitation dataset (1979–2022) extracted from field and satellite observations supplied by the National Oceanic and Atmospheric Administration (NOAA). We applied the areal mean estimation technique, where the average depth of precipitation over a subbasin was estimated from the monthly averages of all cells within the subbasin.

The GRACE and GRACE-FO are twin-satellite missions that provide worldwide temporal variation in terrestrial water storage (TWS)^31^ from 2002 to 2022, expressed as fluctuations in TWS in cm/month (monthly gravity solutions). Three GRACE solutions (RL06) were used and compared to a mean baseline from 2004 to 2009. The three solutions are the CSR-RL06M mascon solutions supplied by the University of Texas—Center for Space Research (UT-CSR) (https://www2.csr.utexas.edu/grace/RL0602_mascons.html) with a grid size of 0.25° × 0.25°^32,33^, the JPL-RL06.1 M mascon solutions from the Jet Propulsion Laboratory (http://podaac.jpl.nasa.gov/grace) with a grid size of 0.5° × 0.5°^34^, and the CSR-RL06SH spherical harmonics solution provided by UT-CSR with resampled grid size of 1° × 1°^35^. The uncertainty of GRACE solutions can be determined by calculating the standard deviation among the three solutions^36,37^. The CSR-RL06M solution was used as the primary dataset (2002–2022), and the standard deviation among the three solutions as the uncertainty^38^.

The time series (1992–2022) for LN surface water levels was derived from the U.S. Department of Agriculture Foreign Agricultural Service (USDA FAS) Global Reservoir and Lake Monitoring (G-REALM) database, where variations in lake height were extracted from satellite altimetry data sets (TOPEX/Poseidon GDR/Jason-1GDR/OSTM/Jason-2 / Jason-3/sentinel-6LR satellite series: ten-day resolution), and reported as monthly lake level height above the mean sea level (https://ipad.fas.usda.gov/cropexplorer/global_reservoir/gr_regional_chart.aspx?regionid=metu&reservoir_name=Nasser&lakeid=000331). The extracted LN time series were correlated with the precipitation time series over the Nile subbasins to examine the correspondence between those two datasets.

We also investigated the response of the downstream areas to the wet periods. Using temporal Landsat 5, Sentinel-2, and Copernicus Digital Elevation Model (DEM) over the Tushka depressions, we calculated the monthly variations in the excess LN water diverted to the TLs during 1998–2003 and 2019–2022. Two imaging datasets were utilized to derive the temporal and spatial variations in the areal extent of TLs’ surface water. Those are the Global Surface Water Explorer dataset (http://global-surface-water.appspot.com)^39^ based on Landsat 5 mission acquisitions, and the Harmonized Sentinel-2 multi-spectral imaging mission supplied by the European Space Agency (ESA) downloaded from the Google Earth Engine^40^. These two datasets classified each pixel as either water or non-water. For each of the TLs, we identified an elevation contour that fits the lake area’s extent for every month throughout the investigated periods. We then calculated the volumes of water subtended between a plane that encloses the identified elevation contour and the DEM of the lake. The elevations were extracted from Copernicus DEM (resolution: 30 m); the latter was downloaded from the Copernicus Open Access Hub website (https://spacedata.copernicus.eu/collections/copernicus-digital-elevation-model).

We monitored the reported stream flow data (1900 to 1999) at Mogren (White Nile), Khartoum (Blue Nile), and Kilo3 (Atbara) stations (Fig. 1c) to estimate the respective contributions to the excess runoff during the 1998–2003 and 2019–2022 events. Specifically, we examined the correspondence between the temporal and spatial variations in precipitation and GRACE_TWS_ over the Nile subbasins and the surface water levels and volumes in LN and the TLs.

### Construct, calibrate, and validate a continuous rainfall-runoff model over the BNS

We constructed, calibrated, and validated a continuous rainfall-runoff model over the BNS^41,42^ that operates based on Hydrologic Response Units (HRUs). The water balance equation, which incorporates daily rainfall, evapotranspiration, runoff, return flow components, and percolation, is applied at the HRU level, each characterized by land use, slope, and soil type. The SWAT model was chosen due to its ability to estimate rainfall-runoff and groundwater recharge over long durations and compatibility with GIS data formats^43,44^.

We used the model to simulate stream flow at the Khartoum station and to compare our estimates to the stream flow at this location. We conducted the following steps to simulate stream flow at the Khartoum gauge station. (1) Delineated the extent of the BNS from the DEM, (2) defined and analyzed the Hydrologic Response Unit (HRUs), each of which has a distinct mix of landscape units, soil, land use, and topography, (3) visualized climate data and outputs of the weather generator to enhance our understanding of climate patterns, trends, and dynamics (e.g., air temperature, either maximum or minimum, precipitation, solar radiation, wind speed, and relative humidity) and to aid in identifying anomalies, incongruent values, or data omissions within the climate data, (4) performed a sensitivity analysis on the parameters to evaluate how variations in parameters affect model outputs, improve model performance, assess uncertainty, rank and select parameters, (5) calibrated the model against average monthly stream flow data (1965–1992) from eight gauging stations (Shamboo, Asosa, Sudan Border, Kessie, Merawi, Roserires, Sennar and Khartoum; Supplementary Fig. 2b) to verify the model correctly reflects the hydrological processes and offer dependable predictions, and (6) validated (1993–2000) the model to assess the model’s performance and to confirm its reliability in predicting watershed responses.

The inputs utilized by the SWAT model incorporate soil types, land use types, Digital Elevation Model (DEM), soil mapping information, and meteorological datasets, comprising air temperature, either maximum or minimum, precipitation, solar radiation, wind speed, and relative humidity. QGIS 3.4 (64-bit) user interface, SWAT^+^ tools, and Microsoft MPI (Message Passing Interface) were used to restore the input files of the SWAT model and discretize the catchment of the Blue Nile into eight subbasins, whose outlets were represented by the gauge stations. The Average Annual Precipitation (AAP) and time series across the BNS were extracted from the GPCP datasets. The data from streamflow gauges along the BNS were acquired from the Egyptian Ministry of Water Resources and Irrigation (MWRI, Cairo) and Global Runoff Data Centre (GRDC; https://portal.grdc.bafg.de). The primary source for land use types data is the United States Geological Survey (USGS) Global Land Cover Characterization (GLCC) dataset (30 m spatial resolution) (https://earthexplorer.usgs.gov/). The soil map utilized in this study was generated by the Food and Agriculture Organization of the United Nations (FAO-UNESCO) (scale 1: 5000000) (https://www.fao.org/soils-portal/data-hub/soil-maps-and-databases/faounesco-soil-map-of-the-world/en/). The extracted data were utilized in conjunction with the Harmonized World Soil Database version 1.2, which integrates updated soil information from diverse regional and national sources globally, along with data from the FAO-UNESCO Soil Map of the World, to improve analysis and comprehension (https://www.fao.org/soils-portal/soil-survey/soil-maps-and-databases/harmonized-world-soil-database-v12/en/). Each of the land use and soil data includes a lookup table that identifies the land use and soil types displayed in the raster map. The soil lookup table provides an additional file containing the soil properties (e.g., bulk density, grain size, water capacity, hydraulic conductivity)^45^. Temporal weather data over the Blue Nile, including average monthly air temperature, either maximum or minimum, solar radiation, wind speed, and relative humidity, were extracted from the Climate Forecast System Reanalysis (CFSR) database (https://climatedataguide.ucar.edu/climate-data/climate-forecast-system-reanalysis-cfsr).

The calibration and validation of the model were conducted using SWAT-CUP2012 software (https://swat.tamu.edu/software/swat-cup/) at eight stations against reported monthly flow rate data to improve the model parameterization. We simulated the flow at each of the eight-gauge stations, starting with the Shambo station and ending at the Khartoum station (Supplementary Fig. 2b). The calibration process included parameter specification by sensitivity analysis, assignment of initial values and permissible ranges for each of the selected sensitive parameters, and final automatic calibration.

The sensitivity analysis was conducted to determine which flow parameters affect the model’s predictions^46–48^. The relative sensitivity of each parameter to others was assessed using t-statistics and p-values. After selecting the sensitive parameters, iterations (number: 2000; using the Latin Hypercube Sampling method) were conducted to automatically adjust the initial values of the selected parameters within the permitted ranges using the semi-automated SUFI2 SWAT algorithms^49^. The t-statistics and p-value were utilized to assess the importance of the relative sensitivity. The t-stat measures sensitivity, where a higher absolute t-statistics suggests higher sensitivity, and the p-value defines the importance level of the sensitivity, where a p-value approaching zero indicates greater significance^50^.

The parameter adjustment during calibration in this study is based on two approaches. The first approach involves substituting a prescribed value (v_) for the parameter from a range of values. The second approach involves making a relative adjustment (‘r_’) to the parameter, where the value from the database of the SWAT model is multiplied by one plus a factor within the specified range^50^. The model was calibrated with flow observation data throughout 1965–1992 and validated over 1993–2000. Seventy percent of the dataset was used for calibration and thirty percent for validation purposes^51^. We were guided by stream flow data availability when selecting the timeframes of calibration and validation. All model runs include a 5-year warm-up period. Multiple statistics were utilized to assess the efficiency of the calibrated SWAT model, including the Nash-Sutcliff Efficiency Coefficient (NSE), Coefficient of Determination (R^2^), and Percentage Bias (PBIAS, %)^26^. NSE values range from 1 to negative infinity, with a value of one indicating an excellent match between simulated and observed flows; a value > 0.5 can be judged as satisfactory, whereas negative NSE values suggest that the time series of the observed values is better than that of the simulated values. R^2^ values span from zero to one, with zero signifying no relationship and one showing an ideal relationship. PBIAS can be positive or negative, but the ideal (optimal) value is zero^26^.

### Downscale and bias-correct GCM outputs over the BNS

The projections of the CCSM4 over the BNS were extracted from http://www.cesm.ucar.edu/models/ccsm4.0/, the HadGEM3 projections from http://www.metoffice.gov.uk/research/modelling-systems/unified-model/climate-models/hadgem3, and GFDL-CM4.0 from https://www.gfdl.noaa.gov/coupled-physical-model-cm4/. The projections were downscaled, bias-corrected, and then utilized to assess how climate change affects the BNS.

The bias correction was performed using the following equation^52,53^.

$${\text{P}}_{{{\text{bias}}\;{\text{correction}}}} \, = \,\left( {{\text{Mean P}}_{{{\text{future}}}} } \right)\, + \,\left( {{\text{Mean P}}_{{\text{o}}} } \right){\,-\,}\left( {{\text{Mean P}}_{{{\text{past}}}} } \right)\, + \,\left( {{\text{Mean P}}`_{{{\text{future}}}} } \right) \, \times \, \left( {{\text{S}}_{{\text{o}}} /{\text{S}}_{{{\text{past}}}} } \right)$$

where Mean P_future_ = a mean of future monthly values for the years 2020 to 2100; Mean P_o_ = a mean monthly value of the observed precipitation data (GPCP) throughout 2006 to 2022; Mean P_past_ = a mean monthly value of the simulated precipitation data (CCSM4, HadGEM3, and GFDL-CM4.0 ) throughout 2006 to 2022; Mean P′_future_ = a difference between monthly value and mean monthly value for the years 2020 to 2100; S_o_ = a standard deviation of monthly observed values throughout 2006 to 2022; and S_past_ = a standard deviation of monthly simulated values throughout 2006 to 2022.

The downscaled and bias-corrected model outputs include the key climatic parameters (air temperature, maximum or minimum, precipitation, solar radiation, wind speed, and relative humidity). Since wind speed is not readily accessible in the CCSM4 model, we have utilized wind speed data from the CESM1-CAM5 (http://www.earthsystemgrid.org) model for this analysis. Both models share the same underlying atmospheric component and were created by the National Center for Atmospheric Research (NCAR). All these parameters were subsequently employed as inputs (data) into the calibrated SWAT model to forecast runoff at the Khartoum station throughout the twenty-first century. Daily data were downloaded, pre-processed by removing invalid or missing data, aggregated into average monthly values, and stored in a format (Comma-Separated Values; CSV) accessible by SWAT.

The Fifth Assessment Report by the Intergovernmental Panel on Climate Change (IPCC) 2014 outlined four potential scenarios based on future greenhouse gas emissions: RCP8.5, RCP6, RCP4.5, and RCP2.6. The higher values indicate higher greenhouse gas emissions, likely resulting in higher global temperatures and more pronounced climate change effects. The radiative forcing of 8.5 W/m^2^ by 2100 due to continued emissions throughout the twenty-first century^54^ makes the RCP8.5 pathway the worst-case scenario. In contrast, the RCP4.5 is considered an intermediate scenario that requires carbon dioxide CO_2_ emissions to decline by mid-century, reaching half the 2050 levels by 2100^54^. We adopted the RCP4.5 and RCP8.5 scenarios to compare intermediate and worst-case scenarios. The default spatial resolution is approximately 1.25^o^ (about 137 km at the equator) and was downscaled to 5 km × 5 km by applying a simple linear interpolation method using Python’s in-built package (https://docs.xarray.dev/en/stable/user-guide/interpolation.html)^55^.

### Use the outputs of down-scaled and bias-corrected GCMs throughout the twenty-first century as inputs to the calibrated rainfall-runoff model

We examined how closely the model outputs of the three GCM models, namely the air temperature, either maximum or minimum, precipitation, wind speed, relative humidity, and solar radiation, corresponded to observations (precipitation from GPCP; all other variables from the CFSR database) throughout a reference period (2006–2022). The outputs, with one exception (precipitation), were similar in magnitude and phase and were utilized directly as inputs to the calibrated SWAT model for 2020 to 2100. Those that differed significantly were bias-corrected before applying them.

### Conduct extreme value and uncertainty analysis of stream flow data in the twentieth and twenty-first centuries.

The extreme value analysis was performed to evaluate precipitation intensity and peak stream flow of extreme events and project any changes in severity from the 20th to the twenty-first centuries. The analysis used observed twentieth century precipitation data, downscaled, bias-corrected twenty-first century precipitation data from each of the three GCMs, and twentieth and twenty-first century stream flow data predicted from the calibrated SWAT model for the BNS. Annual maximums are extracted from each monthly time series data set and used to fit extreme value distributions according to extreme value theory^56^. In this analysis, we evaluate these distributions: The Generalized Extreme Value (GEV), Generalized Logistic (GLO), Generalized Pareto (GP), 3-parameter Lognormal (LN3), Log Pearson Type III (LP3), and Pearson Type III (PE3) distributions. Distributions are fit using the method of L-moments, which is more accurate for predicting the behavior of the tail of distributions^27^. The best distribution was selected based on the ratio of L-moment kurtosis to skewness. The precipitation intensity and peak stream flow are calculated for each return period of 5, 10, 25, 50, 100, and 200 years by evaluating the quantiles (0.8, 0.9, 0.96, 0.98, 0.99, 0.995, respectively) of the fitted distribution. This analysis has been widely used for determining precipitation, drought, and flood depth extremes^57–59^.

This study selected three GCMs (CCSM4, HadGEM3, and GFDL-CM4.0) to represent a range of climate sensitivities encompassing most CMIP5 GCMs when projecting future temperature changes. A random sampling was performed to select annual maximum series (AMS) among the three GCM outputs (e.g., precipitation) to form an ensemble of 500 AMS to quantify the uncertainty of projections due to model variations. Each AMS was fitted to distributions to estimate intensity at all six return periods. The uncertainty range of intensity at each return period is defined by the upper bound at the 95th percentile and the lower bound at the 5th percentile. The uncertainty analysis of peak stream flows for extreme events was repeated in the same way to evaluate the uncertainties of predictions by the SWAT model propagated from climate-forcing data projected by the three GCMs.

## Supplementary Information

Supplementary Information.

## Data Availability

All necessary data to assess the results are included in the manuscript, or the supplementary information and further pertinent datasets are available upon request from the primary author, Ahmed Badawy (a.badawy@wmich.edu).
