# Supplementary material for: Floods of Egypt’s Nile in the 21st century
Source: Sci Rep. 2024 Nov 7;14:27031. doi: 10.1038/s41598-024-77002-8 (PMC11541909; doi:10.1038/s41598-024-77002-8)
Supplement: Supplementary file 1 — Supplementary Information. [file 41598_2024_77002_MOESM1_ESM.docx]

**Floods of Egypt's Nile in the 21st Century**

Table of Contents

**1. Supplementary Figures**……………………………………………………………………..2

1.[1. Supplementary Fig. 1](#_Toc440378970)…………………………………………………………………….2

1.[2. Supplementary Fig. 2……](#_Toc440378971)……………………………………………………………….3

1.[3. Supplementary Fig. 3……](#_Toc440378971)……………………………………………………………….4

**2. Supplementary Tables**………………………………………………………………..….…5

2.[1. Supplementary Table 1](#_Toc440378970)………………………………………………………………..…5

2.[2. Supplementary Table 2](#_Toc440378971)…………………………………………………………….….6

2.3 [Supplementary Table 3……………………………………………………………….…..7](#_Toc440378971)

2.4 [Supplementary Table 4…………………………………………………………….……..7](#_Toc440378971)

**Supplementary Figures**

**Supplementary Fig. 1.** Temporal variations in water storage in the Tushka Lakes (TLs) from October 2019 to December 2022; the red dots represent the maximum water storage for each of the four years.


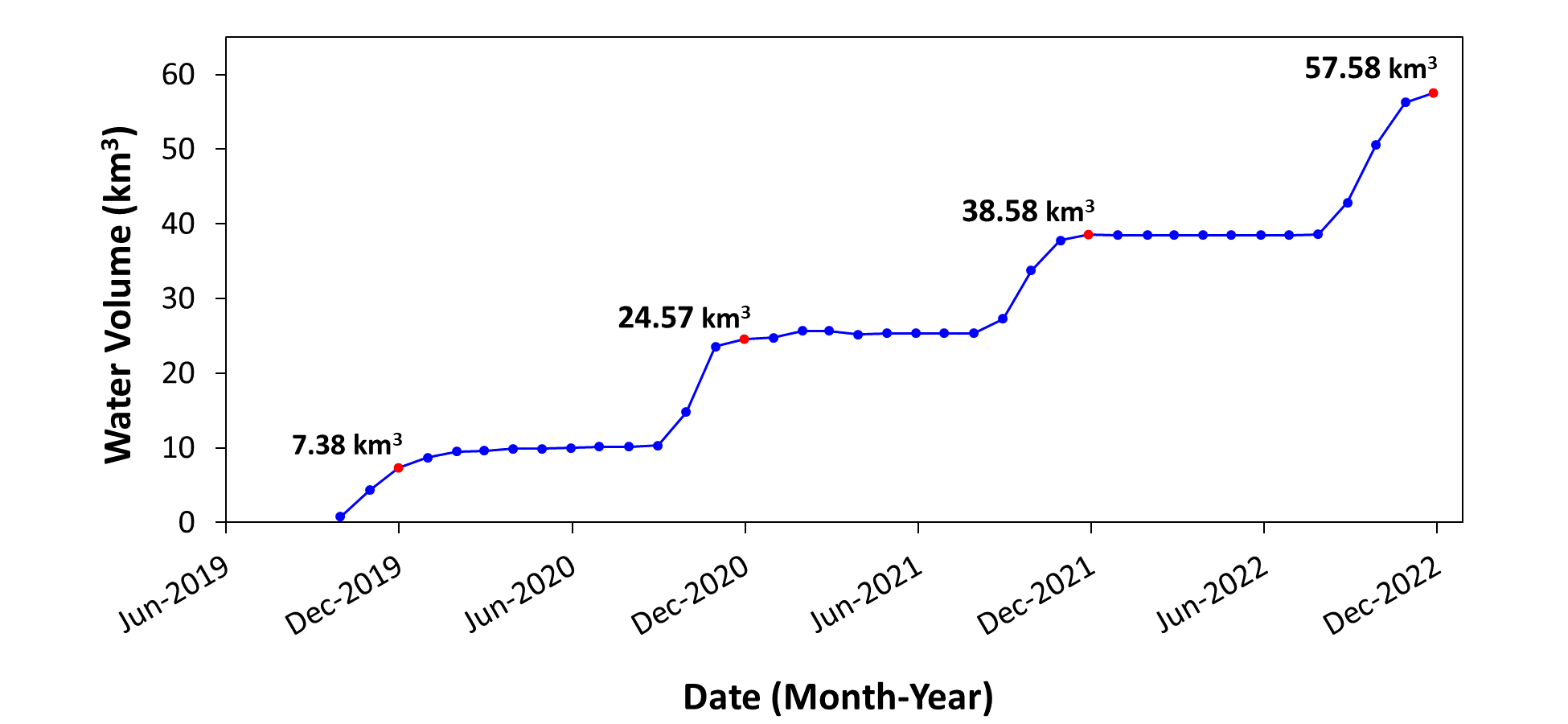


**Supplementary Fig. 2.** Characteristics of the Blue Nile Subbasin, **(a)** Digital Elevation Model, **(b)** stream networks and gauge stations used for SWAT modeling, **(c)** Soil map, and **(d)** Land use map.


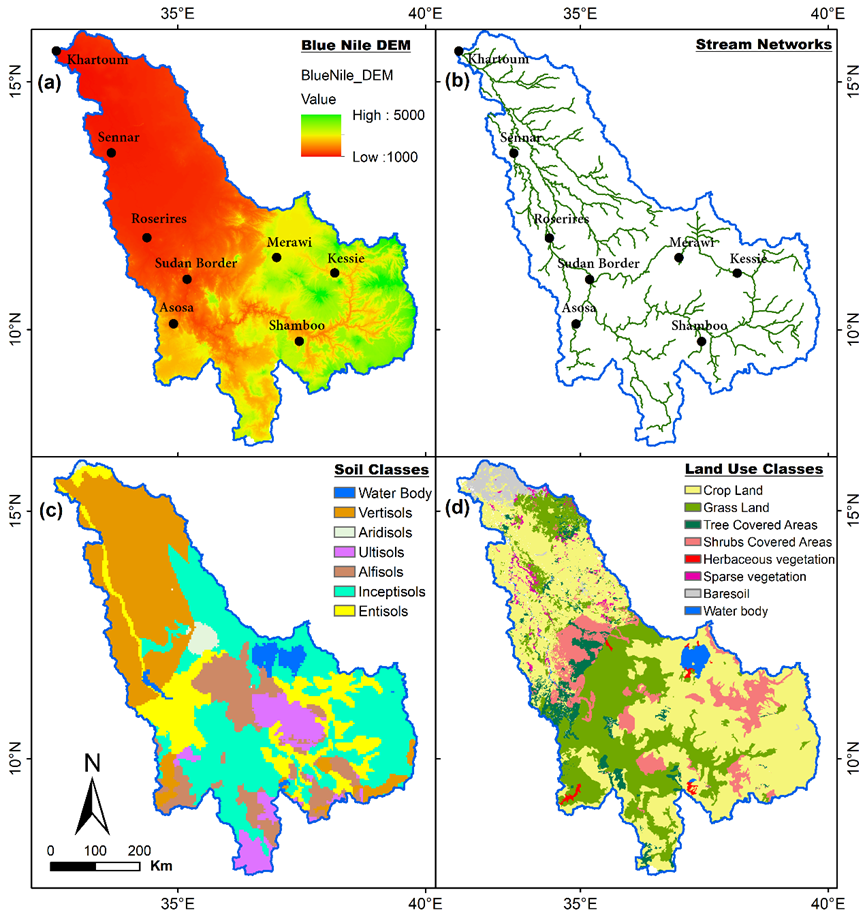


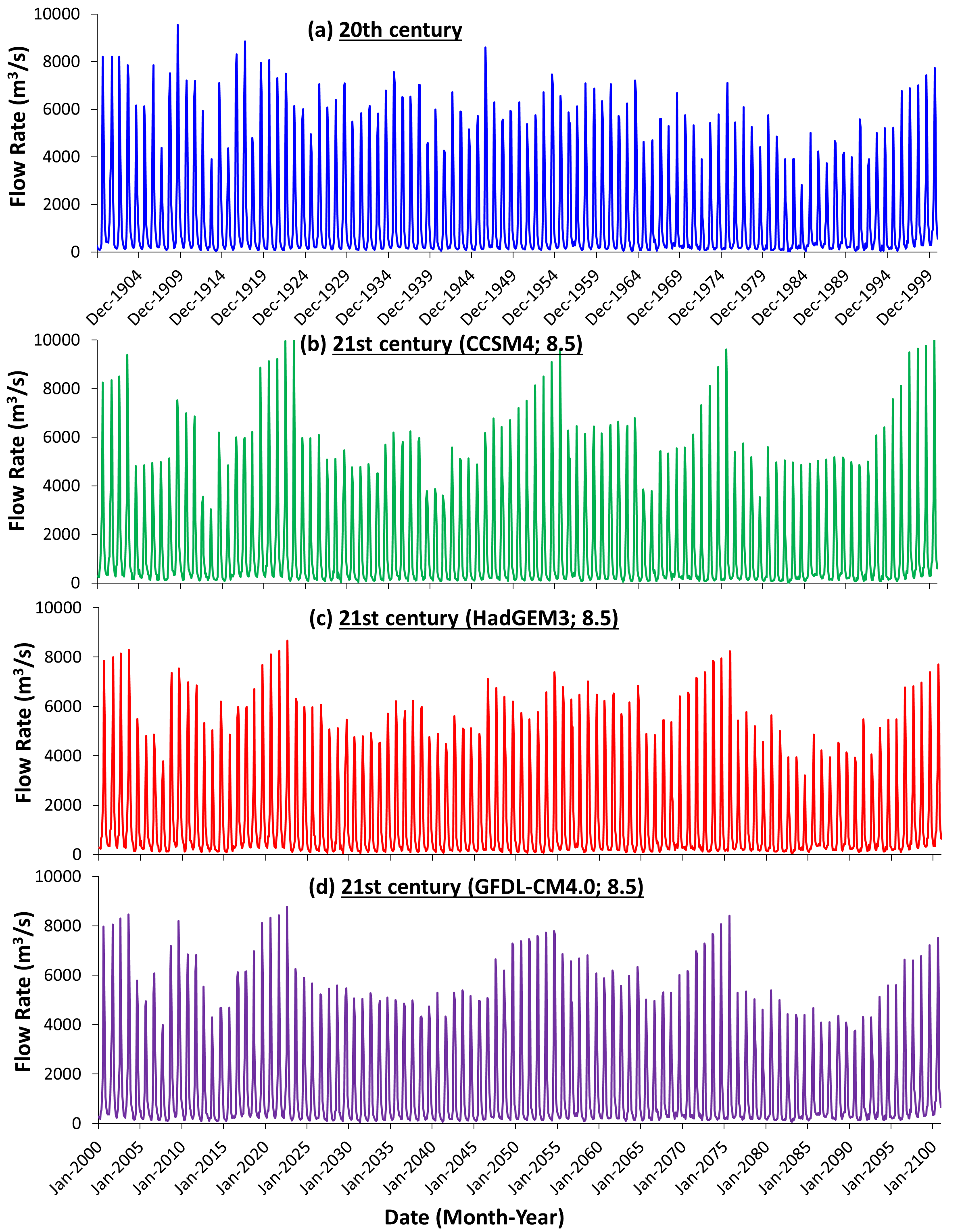


**Supplementary Fig. 3.** Comparison between observed and predicted flow rates from continuous rainfall-runoff model at the Khartoum gauge station in the 20th and 21st centuries based on the RCP8.5 pathway from the CCSM4, HadGEM3, and GFDL-CM4 models. **(a)** observed flow rates during the 20th century. **(b)** predicted flow rates during the 21st century based on the CCSM4 model parameters. **(c)** predicted flow rates during the 21st century based on the HadGEM3 model parameters. **(d)** predicted flow rates during the 21st century based on the GFDL-CM4.0 model parameters.

**Supplementary Table 1**. Temporal variations in water storage in the Tushka Lakes (TLs) from October 2019 to December 2022.

*H, is the Target Height in meters (m),

**V, is Water volume in km^3^,

***TWV, is Total Water Volume in km^3^

**Supplementary Table 2.** Parameter name, description, and parameter space for the calibration parameters of the SWAT model at the Khartoum gauge station.

| **Parameter Name** | **Description** | **Parameter Space** |
| --- | --- | --- |
| v__ALPHA_BF.gw | Baseflow alpha factor (days) | 0.0 – 2.0 |
| r__CN2.mgt | SCS runoff curve number | − 0.50 – 0.50 |
| v__DEP_IMP.hru | Depth to impervious layer in soil profile | 0.0 – 5000 |
| v__SURLAG.bsn | Surface runoff lag time | 0.0001 – 2.0 |
| v__GWQMN.gw | Threshold water depth in the shallow aquifer (mm) | 0.0 – 6000 |
| v__ESCO.hru | Soil evaporation compensation factor | 0.001 – 0.50 |
| r__SLSUBBSN.hru | Average slope length | − 0.70 – 0.90 |
| r__CH_S1.sub | Average slope of tributary channels | − 0.60 – 0.80 |
| r__SOL_Z ().sol | Depth from the soil surface to bottom of the layer | − 0.30 – 0.40 |
| v__CH_N2.rte | Manning's "n" value for the main channel | 0.02 – 0.60 |
| v__CH_N1.sub | Manning "n" value for the tributary channels | 0.004 – 0.70 |
| r__SOL_AWC ().sol | Available water capacity of the soil layer | − 0.30 – 0.40 |
| v__GW_REVAP.gw | Groundwater "revap" coefficient | 0.05 – 0.10 |
| r__CH_S2.rte | The average slope of the main channel | − 0.60 – 0.80 |
| v__OV_N.hru | Manning's "n" value for overland flow | 0.02 – 0.50 |
| r__SOL_K ().sol | Saturated hydraulic conductivity | − 0.40 – 0.40 |

**Supplementary Table 3.** Sensitivity ranking of SWAT model parameters at the Khartoum gauge station.

| **Parameter Name** | **Description** | **t-Stat** | **p-value** | **Rank** |
| --- | --- | --- | --- | --- |
| r__CN2.mgt* | SCS runoff curve number | − 35.0 | 0 | 1 |
| v__DEP_IMP.hru** | Depth to impervious layer in soil profile | 6.91 | 0 | 2 |
| v__CH_N1.sub | Manning "n" value for the tributary channels | 4.85 | 0 | 3 |
| v__ESCO.hru | Soil evaporation compensation factor | − 3.84 | 0 | 4 |
| v__SURLAG.bsn | Surface runoff lag time | − 3.80 | 0 | 5 |
| v__GWQMN.gw | Threshold water depth in the shallow aquifer (mm) | 2.76 | 0 | 6 |
| v__ALPHA_BF.gw | Baseflow alpha factor (days) | 2.51 | 0.04 | 7 |
| r__CH_S2.rte*** | The average slope of the main channel | −1.50 | 0.12 | 8 |
| v__OV_N.hru | Manning's "n" value for overland flow | − 1.35 | 0.14 | 9 |
| r__CH_S1.sub | Average slope of tributary channels | − 1.29 | 0.15 | 10 |
| r__SLSUBBSN.hru | Average slope length | −1.20 | 0.17 | 11 |
| v__CH_N2.rte | Manning's "n" value for the main channel | −1.02 | 0.31 | 12 |
| r__SOL_AWC ().sol | Available water capacity of the soil layer | 0.54 | 0.49 | 13 |
| r__SOL_Z ().sol | Depth from the soil surface to bottom of the layer | 0.28 | 0.82 | 14 |
| r__SOL_K ().sol | Saturated hydraulic conductivity | 0.25 | 0.84 | 15 |
| v__GW_REVAP.gw | Groundwater "revap" coefficient | 0.21 | 0.87 | 16 |

*The file extension (e.g., mgt) denotes the SWAT input file where the parameter is located.

**The qualifier (v) signifies replacing a parameter with a specific value from the provided range.

*** The qualifier (r) denotes a variation in the parameter, where the value retrieved from the SWAT database is multiplied by a factor falling within the designated range.

**Supplementary Table 4.** List of sensitive parameters used for calibration and validation at the Khartoum gauge station.

| **Parameter Name** | **Fitted value** | **Minimum*** | **Maximum**** |
| --- | --- | --- | --- |
| r__CN2.mgt | 0.05 | 35 | 98 |
| v__DEP_IMP.hru | 2323 | 0 | 5000 |
| v__CH_N1.sub | 0.15 | 0.01 | 0.7 |
| v__ESCO.hru | 0.58 | 0 | 1 |
| v__SURLAG.bsn | 33.6 | 0.5 | 50 |
| v__GWQMN.gw | 2100 | 3000 | 5000 |
| v__ALPHA_BF.gw | 0.3 | 0.1 | 0.9 |
| r__CH_S2.rte | 0.3 | 0.1 | 0.5 |

* Minimum SWAT default values.

** Maximum SWAT default values.
